# Miss Martineau’s Marvelous Mesmerization Unmystified

**Published:** 1845-04

**Authors:** 


### MEDICAL INTELLIGENCE.

### MISS MARTINEAU'S MARVELOUS MESMERIZATION UNMYSTIFIED

"A Medical Report of the case of Miss H—— M——," has recently been drawn up and published by Mr. T. M. Greenhow, for general circulation, with "the entire concurrence of the patient,"* [!] "scarcely any one," Mr. G. adds, being really "ignorant of the general character of the precise causes of her continued suffering." The following condensation of this report, which we copy from the *Lancet* of January 4th, will furnish our readers with the prominent details.

"In a letter from Venice, dated June 14th, 1839, Miss M., æt. 37, first communicated to me that, during the preceding year, she had been sensible of a 'great failure of nerve and spirits, and of strength.' Frequently she experienced sharp pain in the uterine region, the catamenia occurring every two or three weeks, and a very irritating discharge, of a brown or yellowish color, taking place in the intervals. The irregular uterine discharges continued, occasionally mixed with clotted blood; and she suffered from many distressing symptoms, evidently arising from uterine irritation; 'inability to stand or walk, aching and weariness of the back, extending down the legs to the heels;' 'tenderness and pain, on pressure, in the left groin, extending by the hip to the back. The spirits became much depressed, and the power of enjoyment was gone.' At the same time, 'a *membranous substance*, like the end of a little finger,' was discovered projecting from the os uteri, described in the following terms, by a friend who accompanied Miss M. on her journey. 'Twice there has been a discharge, similar in color and substance, to blood. Two days ag[illegible] it was found that, from the same passage, (vagina,) was protruding the extrem-

* Miss M. has since stated that Dr. Greenhow was mistaken in supposing he had her entire concurrence to his laying his statement before the public.

ity of a solid substance, totally insensible, of a reddish-brown color, in form resembling the end of a bullock's tongue, with a decided edge or point; it can be pushed back without difficulty or pain, but it falls again.' Either prolapsus uteri or a polypous tumor, of a fibrous nature, was conjectured to be the occasion of these appearances. From Lucerne, July 6, 1839, Miss M. wrote respecting one character of the complaint, (*retroversion of the uterus*,) which took place: 'I cannot walk without injury, but keep my feet laid up, and my knees somewhat raised, as the easiest posture. I began to use the syringe, as you and Dr. Nardo (an Italian physician) recommended; it was a great relief, but, in *three days*, there was *no room for it*, and, on this account, I have never been able to use it since. I discontinued the sponge, finding it irritating, as you say, and it is not now *necessary*.' The sponge was used as a pessary, and the syringe for injecting tepid fluid into the vagina. The occupation of the vagina, by the enlarged and retroverted uterus, I wish to be held in view.

"In July, 1839, Miss M. arrived in Newcastle and placed herself under my care, suffering from the various morbid nervous sensations already described, moderate walking exercise being attended with great inconvenience. The whole symptoms were referable to some derangement of the uterus, which, on examination, was found large, retroverted, and fixed low down in the vagina, the os and cervix uteri occupying the anterior part of the cavity, and the body and fundus of the organ passing horizontally backwards, till the latter approached the sacrum. The enlarged uterus thus occupying the antero-posterior diameter of the pelvis, pressed respectively against the urethra and neck of the bladder and the lower part of the rectum, and this pressure often caused great uneasiness. While the fundus uteri extended backwards, towards the sacrum, the cervix was bent downwards behind the pubes, nearly at a right angle, and, hanging from the lip was a small polypus, which was soon removed, but without alleviation of the symptoms. I was assured, by my patient, that the projecting body which showed itself at Venice was different from, and much larger than this small polypus; and though the os uteri was not dilatable with the finger, and, from its preternatural position, was in a very unfavorable condition for the exclusion of any body contained within the uterus, I for some time hoped that another and larger polypus might again appear. Warm baths and ergot of rye did not promote this object, and soon only appropriate palliatives were employed.

"The tenderness in the left groin was somewhat relieved by leeches, but total rest soon became absolutely necessary, and the nervous discomfort indicated recourse to opiates, used in great moderation, but without much relief being obtained. Subsequently, oppressive retching supervened, and much difficulty in micturition, and in emptying the bowels, attended the pressure of the uterine tumour, as well as the distressing pains frequently extending to the heels. The abdomen became considerably distended, from flatus and other contents; for the uterus could never be felt above the brim of the pelvis, though it doubtless, by pushing the viscera upwards, in some measure, produced a general enlargement of the figure. Not unfrequently, although aperients or injections were used, a gradual accumulation in the bowels took place, giving rise to increased distress, requiring active purgatives. The constant aching in the back rendered it painful to rest upon the sacrum, on the sofa; so a prone position was adopted.

"Little variation took place in the symptoms, or in the affected organ; and, in 1840, I stated the case to Sir Charles M. Clarke, who thought that rest and pallative treatment—the general health being carefully maintained—could alone be depended on; but, in Sept., 1841, Sir Charles visiting this part of the country, after a careful investigation, gave the following opinion in writing, dated September 30th. He says; 'I perfectly agree with you, that the disease was an enlargment of the *body* of the uterus; that the *neck* was perfectly healthy; although the majority of these enlargments of its body do not yield to external applications or to internal remedies; nevertheless, the disorder produces mechanical symptoms only, and *does not lead to any fatal result*, to which the disease of the *neck* does lead. In an instance or two, I *have* known such complaints as Miss M.'s subside; and I would employ for this purpose, the continued external use of iodine ointment,' but which my patient refused to carry into effect. Therefore, I proposed a course of iodide of iron, which, with short intervals, was persevered in till July or August, 1844. The distressing sickness was thus greatly mitigated, the appetite improved, and increased health and mental energy showed itself. The following, in September, 1843, was her own opinion of its effects at that time:—'I suppose I owe my much improved comfort mainly to the pills, (iodide of iron;) indeed it is very great. The pulling and sinking —the mechanical troubles, as one may call them—of course continue; but the almost total absence of sickness, and the striking lessening of the 'distress,' are such a comfort to me!' Occasional examinations of the affected organ took place, but no change could be discovered, excepting the appearance of a membranous substance at the os uteri, which, generally, scarcely protruded beyond its lips, though occasionally described as larger, resembling the appearance observed at Venice, though smaller. It proceeded from the interior of the uterus, and had no attachment to the neck, the finger passing round it on all sides, naturally giving rise to the renewed supposition that the uterus contained a polypous growth, whose separation might be effected by time.

"On April 2d, 1844, I first detected a change in the uterus. The fundus was less fixed, and could be slightly raised from its position. The membranous pedicle remained.

"In June, Miss M. suffered much from indigestion, with loaded bowels. The symptoms proper to the organic affection, especially the distressing pain in the back, were increased; means were used to correct the visceral derangment, and a plaster with belladonna was applied to the sacral region, from which but slight relief was obtained. The unwonted symptoms of indisposition had subsided, when, on June 22d, the mesmeric treatment was commenced, of which a full account has been published in the *Athenæum*, by Miss M. From this time she ceased to be properly under my care, though her accustomed remedies were not yet laid aside; but, on September 4th, I carefully repeated my examination, and found, as on April 2d, the posterior connexions of the uterus less fixed. The retroversion continues, but the fundus, which rests against the rectum and sacrum, feels looser, and admits of being raised to some extent with the finger in vaginam. It is less firm in substance, and the os uteri, to a certain extent, dilatable. Within, and slightly projecting from the os uteri, can be felt *two* substances, which convey to the finger a sensation as if two lumbrici, of moderate size, hung through the mouth of the uterus. These bodies are said, on pressure, occasionally to exude a reddish discharge. Miss M. informed me that the catamenia have resumed their natural course, and that the breasts have increased in bulk. The iodide of iron, and all aperients, have been discontinued, the bowels having lately acted with regularity. The use of opiates has been greatly diminished by enema, and, internally, altogether omitted. The sickness and other gastric inconveniences have ceased; the irritation in the rectum and neck of the bladder is no longer complained of; quietude has succeeded to irritability of the nervous system. 11th.—Comfortable, and opiates greatly diminished; other medicines unnecessary. Been in the garden, on a sofa cushion. 21st.—Reports favorably. Opiate reduced to a very small dose. Walked to the Haven, with no uneasy effects. Oct. 8th.—Can walk two miles and a half. Opiates discontinued. At first, the nights were bad. Last night she slept well, having taken a quantity of brandy and water. No irritation at the neck of the bladder. Some pressure still on rectum; otherwise feels well. Bowels regular.

"Dec. 6th.—Again carefully examined the uterus, which is *less fixed*. The retroversion continues, the fundus still extending towards the sacrum, while the os uteri approaches the pubes. The two membranous pedicles remain hanging out of the os uteri; the health quite good, and the catamenia regular; the nervous pains and irritations all subsided. Renewed habits of activity seem to have greatly contributed to restore the symmetry of the abdomen.

"A glance at the prescriptions employed, except on particular emergencies, during the last three years, will show the error of supposing that Miss M. was in the habit of seeking relief in large and unmeasured doses of opium. (It is unnecessary to copy them in this analysis.)

"Knowing well that no malignant disease of the affected organ existed, I always believed that a time would arrive when my patient would be relieved from most of her distressing symptoms, and released from her long-continued confinement. The catamenial crisis appeared the most probable period, but I did not despair of this happening sooner; though she never willingly listened to my suggestions of the probability of such events, but seemed always best satisfied with admissions that she must ever remain a secluded invalid—an additional symptom of the morbid influence over the nervous system, of the class of diseases in which this case must be included. Oftener than once, I have used the expression, that, probably, before long, Miss M. *would take up her bed and walk*. In this case, the advocates of mesmerism may try to find arguments in support of their opinions. But the experienced practitioner will have little difficulty in bringing the whole into harmony with the well-established laws of human physiology. The condition of the uterus, in December, 1844, is but the natural sequel of the progressive improvement begun in April; and the time had arrived when a new and powerful stimulus only was required, to enable the enthusiastic mind of my patient to shake off the nervous symptoms."---*Med. News.*

